# Human Infection with Highly Pathogenic Avian Influenza Virus (H5N1) in Northern Vietnam, 2004–2005

**DOI:** 10.3201/eid1501.080073

**Published:** 2009-01

**Authors:** Nguyen Duc Hien, Nguyen Hong Ha, Nguyen Tuong Van, Nguyen Thi Minh Ha, Trinh Thi Minh Lien, Nguyen Quoc Thai, Van Dinh Trang, Takuro Shimbo, Yoshimitsu Takahashi, Yasuyuki Kato, Akihiko Kawana, Samu Akita, Koichiro Kudo

**Affiliations:** National Institute of Infectious and Tropical Diseases, Hanoi, Vietnam (N.D. Hien, N.H. Ha, N.T. Van, N.T.M. Ha, T.T.M. Lien, N.Q. Thai, V.D. Trang); International Medical Center of Japan, Tokyo, Japan (T. Shimbo, Y. Takahashi, Y. Kato, A. Kawana, S. Akita, K. Kudo)

**Keywords:** Influenza A virus, H5N1 subtype, respiratory tract infection, antiviral drugs, neuraminidase inhibitors, mortality, research

## Abstract

We performed a retrospective case-series study of patients with influenza A (H5N1) admitted to the National Institute of Infectious and Tropical Diseases in Hanoi, Vietnam, from January 2004 through July 2005 with symptoms of acute respiratory tract infection, a history of high-risk exposure or chest radiographic findings such as pneumonia, and positive findings for A/H5 viral RNA by reverse transcription–PCR. We investigated data from 29 patients (mean age 35.1 years) of whom 7 (24.1%) had died. Mortality rates were 20% (5/25) and 50% (2/4) among patients treated with or without oseltamivir (p = 0.24), respectively, and were 33.3% (5/15) and 14.2% (2/14) among patients treated with and without methylprednisolone (p = 0.39), respectively. After exact logistic regression analysis was adjusted for variation in severity, no significant effectiveness for survival was observed among patients treated with oseltamivir or methylprednisolone.

## Abstract 2

Treatment with oseltamivir or methylprednisolone was not effective, and 7 of 29 patients died.

Human infection with the highly pathogenic avian influenza A virus (H5N1) was discovered in Hong Kong Special Administrative Region, People’s Republic of China, in 1997 (*1*–*3*). It has since been identified in other countries, primarily in Southeast Asia. Among 100 confirmed infected patients, 46 have died in Vietnam since 2003 (*4*,*5*).

Severe viral pneumonia accompanied by diffuse alveolar damage develops in patients infected with influenza virus (H5N1) (*6*). High viral load causes intense cytokine reactions and inflammation (*7*). Clinical factors that might be associated with severity include age, delayed consultation, lower respiratory tract lesions, and leukopenia (*4*,*8*–*10*). However, few cases have reported which factors, including patient management, affect outcomes. Our study reviews the clinical courses of patients treated in Hanoi, Vietnam, and investigates the association between clinical findings and survival.

The effects of oseltamivir and other neuraminidase inhibitors have been demonstrated in experimental models (*11*–*13*), but their outcomes in humans have not been verified. Randomized controlled trials would be optimal for investigating the effectiveness of oseltamivir compared with placebo, but they are not an option because of ethical issues. Therefore, this issue can only be addressed through observational studies. Despite limited empirical evidence, the World Health Organization (WHO) reported that oseltamivir improved survival (*14*) and recommended treatment with oseltamivir because of high mortality rates associated with influenza A virus (*14*,*15*). Patients from northern Vietnam are described in detail.

## Methods

We investigated patients infected with influenza A virus (H5N1) who were referred to the National Institute of Infectious and Tropical Diseases in Hanoi, Vietnam, from other local hospitals from January 2004 through July 2005. Pediatric patients were admitted to another institution in Hanoi and were excluded from the present study. A WHO inspection team at the National Institute for Hygiene and Epidemiology in Hanoi virologically confirmed H5N1 subtype infection in the patients by using a reverse transcription–PCR (RT-PCR) for influenza A/H5. We investigated only patients with H5N1 subtype infection determined from symptoms of acute respiratory tract infection, a history of high-risk exposure, or chest radiographic findings such as pneumonia. All patients were reported to WHO as having confirmed infection with avian influenza virus (H5N1). We excluded other patients with positive RT-PCR results because of reasons described below. The study was reviewed and approved by the ethics committees at the International Medical Center of Japan and the National Institute of Infectious and Tropical Diseases in Vietnam.

Data were obtained for general characteristics, history of high-risk exposure, medical history, symptoms, signs, microbiologic and biochemical test results, chest radiographic findings, treatment strategies, and outcomes from medical records from April through October 2006.

We investigated associations between clinical findings and survival by using univariate analysis. Initial laboratory and chest radiographic findings after hospitalization were recorded in the medical charts and used. The relationship between survival and treatment with oseltamivir or methylprednisolone was investigated by adjusting for factors related to severity in an exact logistic regression analysis, which is appropriate for small amounts or unbalanced binary data (*16*). Because the study cohort was small, deaths were few and overfitting was possible (*17*,*18*); only 1 covariate could be added for adjustment into the logistic regression model. Therefore, we used leukocyte counts, platelet counts, aspartate aminotransferase (AST) levels, and urea nitrogen levels as an adjustment for severity because these values are associated with reported outcomes (*14*). Also, many missing observations prevented adjustment using albumin levels. Data were analyzed by using the Wilcoxon test, χ^2^ test, and Fisher exact test when appropriate and the statistical package SAS version 8.2 (SAS Institute, Cary, NC, USA).

## Results

Among 41 patients who were hospitalized from January 2004 through July 2005 and had positive RT-PCR results, 12 were excluded from the study (3 patients whose medical records were unavailable; 2 patients related to persons with confirmed H5N1 subtype pneumonia who were asymptomatic, positive for viral RNA, and treated with prophylactic oseltamivir; and 7 patients who had some illnesses, particularly respiratory diseases, which complicated interpretation of the clinical course or chest radiographic findings). We therefore studied 29 patients with clinically and virologically confirmed influenza A (H5N1) infection.

Table 1 shows the general characteristics of the patients, and the Figure shows the clinical course from onset of disease to hospitalization and discharge. Patients ranged in age from 14 to 67 years and with a mean age of 35.1 years. A total of 25 patients were given 150 mg/day of oseltamivir, and 15 were treated with methylprednisolone (initial dose 40–160 mg/day, median dose 80 mg/day). Seven (24.1%) of the 29 patients died. No significant associations were found between mortality rates and age (p = 0.57), sex (p = 0.68), history of high-risk exposure (contact with poultry [p = 1.00], contact with sick poultry [p = 1.00], and contact with sick poultry or persons [p = 1.00]). Three of 6 patients from a family infected with H5N1 subtype died, and 4 of 23 patients without such an association died (p = 0.13). Duration between onset of disease and hospitalization was not associated with higher mortality rates (p = 0.98).

**Table 1:** Characteristics of 29 patients infected with highly pathogenic avian influenza virus (H5N1), northern Vietnam, 2004–2005*

Characteristic	Value
Age, y, mean ± SD	35.1 ± 14.4
M:F sex (%)	15:14 (52:48)
High-risk exposure, no. (%)†	
Poultry	19 (65.5)
Sick poultry	12 (41.4)
Family infected with H5N1 virus subtype	6 (20.7)
Sick poultry or person	15 (51.7)
Hospitalization after disease onset, median, d (IQR)	6 (4–8)
Hospital stay, median, d (IQR)	14 (9–17)
Treated with oseltamivir, no. (%)	25 (86.2)
Began treatment with oseltamivir after disease onset, median, d (IQR)	7 (5–10)
Treated with methylprednisolone, no. (%)	15 (51.7)
Died, no. (%)	7(24.1)

*IQR, interquartile range. †Poultry, a history of exposure to sick or healthy poultry; sick poultry or person, a history of exposure to sick poultry or a family infected with avian influenza (H5N1).

**Figure:**
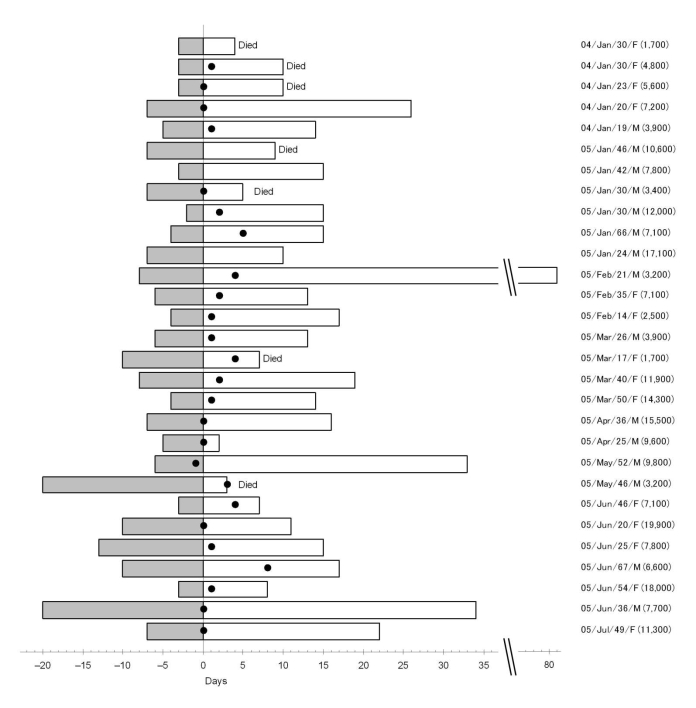
Clinical course of 29 patients infected with highly pathogenic avian influenza virus (H5N1), northern Vietnam, 2004–2005. Zero days on horizontal axis represent days of hospitalization at the National Institute of Infectious and Tropical Diseases. Shaded bars, days between disease onset and hospitalization; open bars, days between hospitalization and discharge; dots, start of oseltamivir treatment. Information on the right shows date of hospitalization, age in years, sex, and leukocyte count per microliter.

Table 2 shows initial laboratory findings at hospitalization. Leukopenia (neutropenia), thrombocytopenia, hypoalbuminemia, and increased AST and urea nitrogen levels were associated with increased deaths.

**Table 2:** Initial laboratory and chest radiographic results for 29 patients infected with highly pathogenic avian influenza virus (H5N1), northern Vietnam, 2004–2005*

Characteristic	Survived, median (interquartile range), n = 22	Died, median (interquartile range), n = 7	p value
Leukocytes, × 10^3^/μL	7.8 (7.1–12.0)	3.4 (1.7–5.6)†	0.0093
Neutrophils, ×10^3^/μL	6.8 (4.8–9.9)	2.3 (1.1–3.8)†	0.0101
Hemoglobin, g/L	130 (107–137)	121 (103–138)	0.6102
Platelets, × 10^3^/μL	214 (181–284)	86 (38–139)†	0.0101
Albumin, g/L	34.5 (31.2–35.1)	21.7 (10.4–29.4)†	0.0265
AST, U/L	45 (28–69)	327 (77–352)†	0.0077
Total bilirubin, μmol/L	10.3 (7.6–16.8)	11.4 (7.0–27.1)	0.7921
Urea nitrogen, mmol/L	4.5 (3.4–5.5)	9 (3.4–14.3)†	0.0462
Initial chest radiographic findings‡			
No or slight lesion	7	1	0.6510
Moderate lesion	10	3	
Severe lesion	5	3	

*Not all laboratory findings and chest radiographic results were available. Results were derived from the following numbers of patients: complete blood count, 29; albumin 12; AST, 25; total bilirubin, 17; urea nitrogen, 27. AST, aspartate aminotransferase. †p<0.05, by Wilcoxon test or Fisher exact test. ‡No or slight lesion, no lesion or localized (occupying less than one third of unilateral lung fields) in 1 lung; moderate, diffuse in 1 lung or localized but evident in both lungs; severe, diffuse in both lungs.

Five (20.0%) of the 25 patients treated with oseltamivir died, as did 2 (50.0%) of 4 who were not treated (odds ratio 0.25, 95% confidence interval [CI] 0.03–2.24, p = 0.24). To adjust for variation in disease severity among patients, exact logistic regression was performed by using leukocyte counts, platelet counts, AST levels, and urea nitrogen levels. Adjusted odds ratios for deaths among patients treated with oseltamivir were 0.15 (95% CI 0.00–2.57, p = 0.19), 0.16 (95% CI 0.00–2.23, p = 0.17), 0.54 (95% CI 0.02–11.85, p = 1.00), and 0.28 (95% CI 0.01–5.16, p = 0.55), respectively, for the 4 adjustments for disease severity.

The time between the onset of symptoms and initiation of treatment with oseltamivir varied (Table 1, Figure). The mortality rates were 20% (3/15) and 20% (2/10) when treatment with oseltamivir was started within and after 7 days of disease onset.

Methylprednisolone was given to 15 of 29 patients. Five (33.3%) of these 15 patients died, and 2 (14.3%) of 14 patients who were not given this drug died (odds ratio 3.0, 95% CI 0.48–18.93, p = 0.39). Exact logistic regression after adjustment for severity by using leukocyte counts, platelet counts, AST levels, or urea nitrogen levels showed odds ratios for deaths among patients treated with methylprednisolone of 0.74 (95% CI 0.00–9.57, p = 0.82), 1.82 (95% CI 0.18–25.48, p = 0.89), 1.14 (95% CI 0.07–18.92, p = 1.00), and 2.43 (95% CI 0.28–31.69, p = 0.61), respectively.

Thirteen patients were treated with oseltamivir and methylprednisolone. The regression model that included these 2 drugs and interactions did not show effectiveness of either drug.

## Discussion

The overall mortality rate of 24.1% in this study was lower than rates in previous studies and WHO reports. Table 3 summarizes the characteristics of patients from previous studies. Patients in the present study were older because pediatric patients were excluded because of treatment elsewhere. WHO has reported that the mortality rate of 73% for infection with H5NI subtype is highest in persons 10–19 years of age, and that patients 20–39 years of age account for >60% of the deaths (*22*). The expected mortality rate would be 51.8% if our case-patients had the same age-specific mortality rate as in a WHO report (*14*). The lower mortality rate in our study could not be explained by an age difference. The relatively high leukocyte count and factors related to outcomes suggest that a reasonably large number of mildly infected patients might have been included, although chest radiographs showed variable progression in lesions from mild to severe.

**Table 3:** Comparison of studies of influenza A virus (H5N1)–infected patients*

Author (reference)	Year	Patient age, y, mean ± SD	Leukocyte count, median, × 10^3^/μL (IQR)	Hospitalization after disease onset, median, d (IQR)	Outcome	
No. alive	No. died
Present study	2009	35.1 ± 14.4	7.2 (3.9–11.3)	6 (3.5–8)	22	7
Yuen at al (*8*). †	1998	17.6 ± 20.4	5.6 (2.47–10.7)	3 (2–4)	5	5
Tran et al. (*4*)	2004	13.7 ± 6.4	2.1 (1.9–2.8)	6 (5–7)	2	8
Chotpitayasunondh et al. (*9*)	2005	22.0 ± 21.4	4.43 (2.75–5.64)	NR	4	8
Oner et al. (*19*)	2006	10.1 ± 4.0	3.8 (1.8–5.75)	6 (4–7)	4	4
Kandun et al. (*20*)	2007	15.4 ± 14.9	3.59 (2.605–6.3)	7 (5–7)	4	4
Buchy et al. (*21*)	2007	16.0 ± 9.9	4.2 (3.6–4.7)	6 (5–7)	0	6

*IQR, interquartile range; NR, not reported. †Outcomes of 2 patients were not reported.

Persons who died were concentrated in the early period of the study, especially in 2004. Virus genotype and load data could provide useful information on pathogenesis and outcome. However, these data were not available.

Factors affecting outcome were leukocyte and platelet counts, and albumin, ALT, and urea nitrogen levels. Results were consistent with previous findings (*14*) and suggested that outcome is related to lesions in several organs.

Although the mortality rate was lower among patients treated with oseltamivir, differences were not significant. Exact logistic regression after adjustments for laboratory results yielded an odds ratio of 0.15–0.54 for death. The small number of patients prevented valid adjustment, and confounding factors might not have been sufficiently eliminated. A larger patient cohort should be able to adjust for severity of disease.

If one considers the possibility of confounding factors, the reason oseltamivir was not prescribed should be investigated. If oseltamivir was withheld from patients with severe infections and administered only to those with milder symptoms, the drug would apparently be more effective. Among 4 patients who were not prescribed oseltamivir, initial RT-PCR results were negative for 1 patient, who subsequently died. Oseltamivir was unavailable for treatment of another patient who died. The other 2 surviving patients were not prescribed oseltamivir because their chest radiographs showed only minimal lesions. Therefore, withholding oseltamivir was not associated with disease severity.

Higher doses of oseltamivir or longer drug administration have improved outcomes in animal models (*23*,*24*). Because all patients in our study were given oseltamivir at a dose of 150 mg/day, we could not investigate the effect of a higher dose.

Mortality rates were higher in patients treated with methylprednisolone than in those not treated with this drug. This finding can be explained by disease severity because severely ill patients were more likely to be given methylprednisolone. However, even after we adjusted for this confounding effect, no beneficial effect of methylprednisolone was observed. Further, an experimental model has recently raised doubt about the effect of cytokine suppression (*25*).

Our study described patients infected with influenza A virus (H5N1) in Hanoi, Vietnam. These patients had lower mortality rates than those reported in other studies. The reason for the low mortality rate could not be investigated thoroughly without virologic information. Oseltamivir was prescribed in 25 of 29 patients, and their mortality rate was apparently decreased, although the patient cohort was too small to generate sufficient statistical power. In addition, since our study was an observational study, these findings might have been influenced by confounding factors. Further detailed observations from a larger number of patients are required.
